# Assessing Temperature Distribution inside Commercial Stacked Cage Broiler Houses in Winter

**DOI:** 10.3390/ani14182638

**Published:** 2024-09-11

**Authors:** Senzhong Deng, Zonggang Li, Yongxiang Wei, Yang Wang, Baoming Li, Weichao Zheng

**Affiliations:** 1 Department of Agricultural Structure and Environmental Engineering, College of Water Resources and Civil Engineering, China Agricultural University, Beijing 100083, China; szdeng14@cau.edu.cn (S.D.);; 2 Beijing Key Laboratory of Detection Technology for Animal-Derived Food Safety, College of Veterinary Medicine, China Agricultural University, Beijing 100193, China; 3 Department of Animal Genetics and Breeding, College of Animal Science and Technology, China Agricultural University, Beijing 100193, China; 4 Key Laboratory of Agricultural Engineering in Structure and Environment, Ministry of Agriculture and Rural Affairs, Beijing 100083, China; 5 Beijing Engineering Research Center on Animal Healthy Environment, Beijing 100083, China

**Keywords:** broiler, climate, thermal environment, pressure difference, ventilation

## Abstract

**Simple Summary:**

A suitable environment inside broiler houses is crucial to broiler health, welfare, and productivity. High stocking density can easily lead to uneven temperature conditions, which may cause broilers to experience cold or heat stress. Therefore, the objectives of this article were to assess the temperature uniformity inside a commercial broiler barn and to investigate influencing factors on the temperature difference. In this study, temperature, wind velocity, and differential pressure sensors were installed in a commercial stacked-deck cage broiler house for continuously monitoring the environment. Results indicate that the maximum temperature difference increased from 1.85 °C to 6.43 °C, while the daily fluctuation increased from 2.27 °C to 5.80 °C. The lateral temperature distribution is the main reason for the poor overall temperature uniformity and stability. In the lateral direction, the temperature distribution changes periodically, with the maximum temperature difference varying from 1.98 °C to −2.14 °C (− indicating high-temperature location variation) within one day. This variation was significantly influenced by solar radiation and wind speed, which can lead to poor performance and higher mortality rates in broilers. Therefore, it is crucial to consider the influences of solar radiation and wind on the indoor environment when designing and managing chicken houses.

**Abstract:**

The temperature inside broiler houses is crucial to broiler health, welfare, and productivity. High stocking density in broiler houses can easily lead to nonuniform temperature conditions, which would cause broilers to suffer cold and heat stress. It is essential to assess the temperature distribution inside broiler houses and investigate the factors that affect temperature uniformity. Therefore, in this study, temperature, wind velocity, and differential pressure were monitored in the aisle, at the sidewall inlet, and outside the sidewalls of a commercial stacked-deck cage broiler house in Northeast China aiming to continuously monitor the temperature throughout the entire fattening period. Results show that the maximum temperature difference increased from 1.85 °C to 6.43 °C, while the daily fluctuation increased from 2.27 °C to 5.80 °C. The highest temperature was consistently recorded at the side of the exhaust fans (*p* < 0.001) in the longitudinal direction. In the lateral direction, the temperature difference varies periodically with solar radiation. The average temperature at the southern location (23.58 ± 1.97 °C), which faces the sun, was higher than that at the northern location (23.35 ± 1.38 °C), which is in the shade, during periods of solar radiation (*p* < 0.001) at the last testing period. However, without solar radiation, the northern location recorded a warmer temperature (23.19 ± 1.41 °C) compared to the southern location (22.30 ± 1.67 °C) (*p* < 0.001). The lateral temperature differences are strongly positively correlated with solar radiation and wind speed (*p* < 0.001). In conclusion, the inside temperature nonuniformity and fluctuation increased as the broiler age increased, which affected the production performance of broilers. Nonuniform solar radiation and wind speed can lead to differences in the inlet temperature and air volume between both sidewalls, thereby affecting the uniformity of the lateral temperature inside the house.

## 1. Introduction

The broiler breeding industry is a vital component of animal husbandry. In recent years, driven by the rising demand for chicken meat products and the need to enhance meat production efficiency, the chicken industry has gradually moved toward intensive and large-scale development in China. Broiler production in China has increasingly relied on stacked-deck cage-rearing systems, which offer large feeding capacities and high space utilization [1]. Due to the intensive rearing practices in broiler houses, the indoor environment has become a focal point of attention in the farming process [2,3]. Providing an appropriate living environment inside the broiler house, particularly the interior air temperature, is one of the most crucial factors in improving broiler production, which seeks to achieve high productivity in a relatively small space and time [4].

Temperature affects the physiological functions of poultry birds at any stage of life, which affects poultry health and production performance [5]. Due to the lack of sweat glands, abundant feathers, and fast metabolic rates, neonatal broiler chickens are highly susceptible to low temperatures given the immaturity of their thermogenic organs [6]. The exposure of chicks to a cold environment could have a carry-over negative effect on body mass, feed intake, and growth performance [7]. Low temperatures can adversely impact the health of broilers, leading to an inflammatory response and apoptosis [8,9]. High temperatures could significantly impact the physiological function of broilers, resulting in metabolic disturbances, immune depression, and endocrine impairment, ultimately affecting the production performance and rearing efficiency of broiler chickens [10,11]. For example, broilers subjected to heat stress conditions had a 16.4% reduced feed intake, 32.6% body weight, and a 25.6% higher feed consumption ratio [12]. High stocking density in broiler houses can easily lead to uneven distribution of environmental temperature, resulting in some broilers suffering cold stress while others suffer heat stress, which leads to reduced feed intake, feed consumption, and body weight [13,14]. Therefore, ensuring a uniform and comfortable environment in the chicken house is of utmost importance.

The existence of temperature differences inside poultry houses with tunnel ventilation is a common phenomenon in recent research, especially in winter. The highest air temperature was usually found near the exhaust fan, whereas the lowest air temperature was observed near the front wall in the long [15,16]. The temperature at higher heights was warmer than that at lower heights, which was caused by the accumulation of heat in higher spaces [17]. This research on the thermal environment in broiler houses during winter is usually concentrated on studies conducted under ground-breeding system conditions [18,19,20]. Other studies have focused on simulating the broiler house environment through computational fluid dynamics (CFD) techniques, which have limitations in reflecting the dynamic environment throughout the entire rearing period [15,21,22]. Therefore, the assessment of the temperature distribution of the broiler house using stacked cage rearing systems throughout the entire rearing period is of utmost importance for providing a reference for the management of the poultry house environment.

The environmental control system of the poultry house is important for providing a suitable indoor temperature [23]. Proper ventilation helps to dissipate excess air moisture and pollution gases while also improving temperature uniformity [24]. Ma et al. explored improving the indoor thermal environment by optimizing the exhaust fan layout of tunnel ventilation [22]. Wang et al. designed a new ventilation system in which evaporative cooling pads were installed on both sidewalls and exhaust fans located on a gable wall, which mitigated air temperature variations and improved uniformity in poultry houses [25]. Researchers primarily focused on the optimization of ventilation system structures, but the overlooked temperature inside was also impacted by climate, including solar radiation and wind. Solar radiation is an important factor in the building’s thermal environment influencing the heat transfer through the roof and sidewalls of the poultry house [26]. The wind impacts the static pressure difference of the slot opening, which is the driving force for air intake. Therefore, static pressure may influence the ventilation performance of poultry houses [27]. The literature primarily focuses on the impact of the aforementioned parameters on the overall environmental conditions within poultry houses [28,29]. The effects of solar radiation and wind on temperature differences inside broiler houses have not been investigated.

The objectives of this article were (1) to assess the uniformity of interior air temperature over the entire growing period inside a commercial broiler barn with a stack cage housing system in winter and (2) to analyze the monitoring data for studying the effects of solar radiation and wind speed on the temperature difference inside the broiler house.

## 2. Materials and Methods

### 2.1. Field Experimental Site

**Figure 1:**
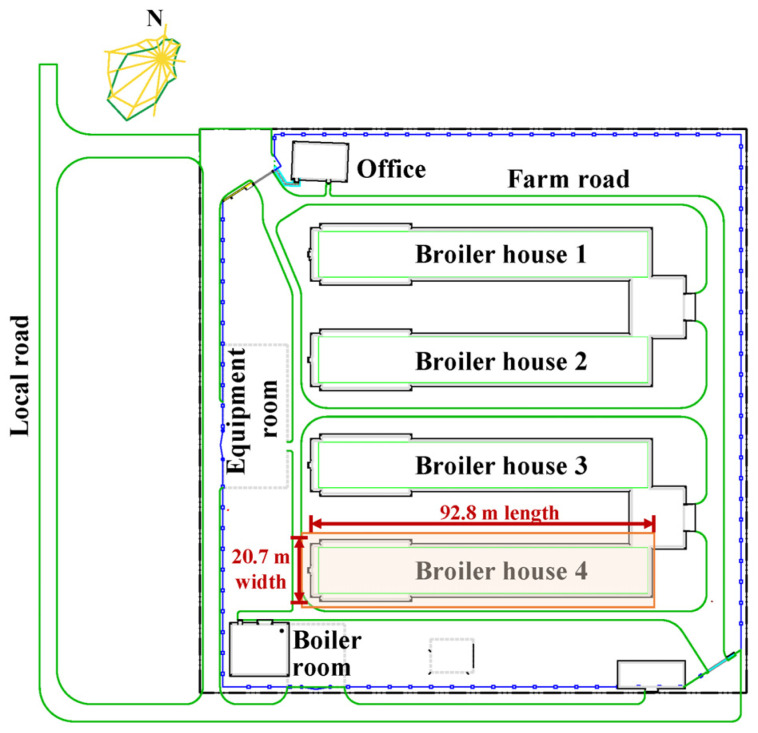
Layout of the experimental farm and experiment location (Broiler house 4).

This study was conducted in a commercial broiler farm situated in Northeast China (44°50′23.60″ N, 126°31′58.80″ E); this area has a continental climate and experiences south winds throughout the year. The farm consists of four broiler houses, each oriented in the east–west direction, with dimensions of 92.8 m in length, 20.7 m in width, and a height of 6.5 m (Figure 1). Within these structures, an approximate total of 312,000 broilers (Arbor Acres) were housed with 78,000 broilers in each house using seven rows of four-tier conventional cages.

**Figure 2:**
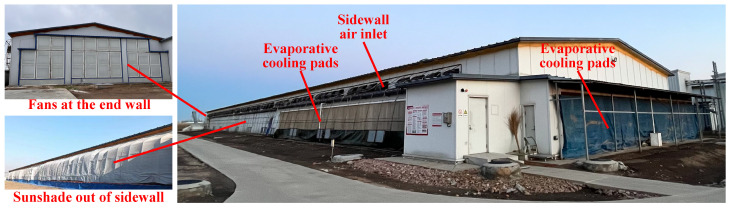
Field conditions of the testing broiler house.

**Figure 3:**
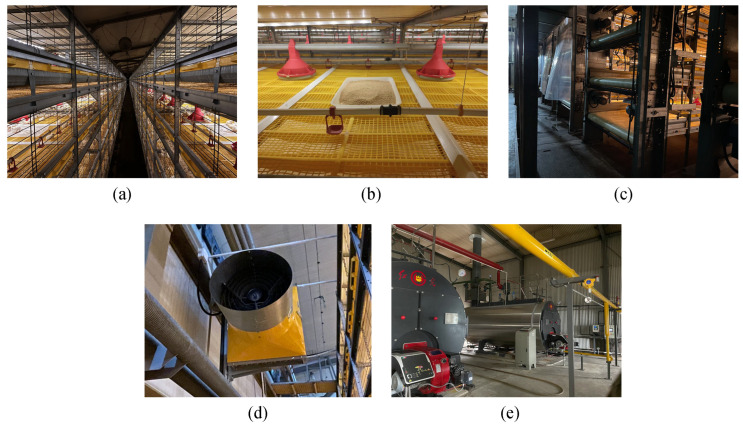
Broiler fattening system: (**a**) stacked-deck cage system, (**b**) the feeding and drinking system, (**c**) conveyor belt manure removal system, (**d**) air–water heat exchanger, and (**e**) boiler of the farm.

The environmental conditions were monitored in house 4, the external envelopes of which were constructed from foamed polyurethane material. The thicknesses of the wall and roof insulation were 100 mm and 120 mm, with thermal conductivities of 0.18 W/(m^2^·K) and 0.21 W/(m^2^·K), respectively. A sunshade net (6 Needle) was covered over both sidewalls of broiler houses to reduce the impact on sidewall inlet ventilation of wind velocity outside in winter (Figure 2). As shown in Figure 3a, the broilers are raised in conventional stacked-deck cages, with an average of 95 broilers per cage. The relevant parameters of the cages are listed in Table 1. Each cage is equipped with 14 nipple drinkers and 2 feeders (Figure 3b). Additionally, a conveyor belt is installed beneath each layer of cages to automatically remove the manure produced by the broilers (Figure 3c).

The barn uses a typical tunnel-ventilation system for environmental control, allowing fresh air to enter through end-wall evaporative cooling pads in hot and mild weather and through sidewall inlets during cold weather, with the air being exhausted by a fan. As shown in Figure 2, the cooling pads are installed on the gable wall and both sidewalls at one end of the building, featuring a thickness of 150 mm and areas of 23.4 m^2^ and 42.4 m^2^, respectively. Each sidewall is equipped with 50 air inlets, each measuring 0.82 m × 0.33 m and positioned 3.4 m above the ground. At the opposite end of the building, there are 24 fans with shutters and cones (EM52, 1.42 m diameter, 40,000 m^3^/h, Munters, Kista, Stocksund, Sweden), along with 2 smaller capacity fans (EM36, 1.1 m diameter, 20,000 m^3^/h, Munters, Kista, Stocksund, Sweden). Eight air–water heat exchangers (Heat-X Type-4H, fan capacity 7500 m^3^/h, Reventa GmbH, Kista, Stocksund, Sweden) were installed to maintain the recommended air temperature in winter (Figure 3d). The power of the air–water heat exchanger is related to the inlet medium flow temperature, return temperature, and the temperature of air intake. The equipment manual indicates that when the inlet water temperature, outlet water temperature, and air temperature are 80 °C, 60 °C, and 20 °C, respectively, the power of the heat exchanger is 95 kW. The heat source for the heat exchanger is a natural gas boiler (WNS2.8, 2.8 MW power, Harbin Hongguang Boiler Group Co., Ltd., Harbin, China) that generates hot water through the combustion of natural gas (Figure 3e). This hot water is then sent to the heat exchanger to warm the air inside the chicken house. Additionally, the hot water produced by the natural gas boiler is supplied not only to four chicken houses but also to the living areas. The environmental control equipment inside the barn, including fans, evaporative pads, sidewall air inlet, and air–water heat exchangers, are controlled by an environmental controller (Hotraco ACA-1N, Hotraco Agri, Hegelsom, The Netherlands).

The broiler chickens were reared for a total of 38 days with the target temperature decreased from 35.0 °C to 20.2 °C. During the study, the light regime was variable, based on the age of the broiler, as follows: For the first 3 days and from Day 32 to Day 38, the lights remained on during the daytime without dimming and were turned off for 1–2 h at 23:00. From Day 4 to Day 31 of the broiler’s age, the lights were dimmed for 1–3 h starting at 11:00 during the daytime. In the evening, the lights were turned off for 2 h from 19:00 to 21:00. The broilers were fed three times daily. In wither, the barn ventilation operated in a mode in which fresh air entered the barn through slot openings along both sidewalls, and barn air was exhausted through tunnel fans at the end wall. The fans are not operated continuously in cold weather, and the interval timer permits intermittent operation of minimum ventilation fans to reduce convection heat dissipation. During the rearing period, the number of open ventilation fans was increased from 1 to 4. The designed minimum ventilation rate of the barn was approximately 0.04 to 0.77 m^3^/h per bird as the chicks grew. The ventilation rate, heating temperature, and cooling temperature for broiler chickens, as provided by the onsite manager, are shown in Table 2. To maintain a suitable temperature, whenever any temperature sensor inside the poultry house fell below the heat temperature, the heater was activated. From age 28 to age 38 of broiler, the heater was deactivated because of sufficient heat production by the broilers.

### 2.2. Environmental Monitoring

**Figure 4:**
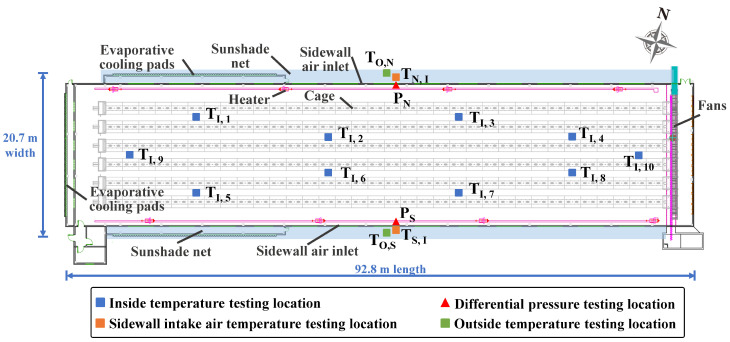
Broiler house cross-sectional view demonstrating the air inlet, sampling locations, and sunshade net on the exterior wall.

**Figure 5:**
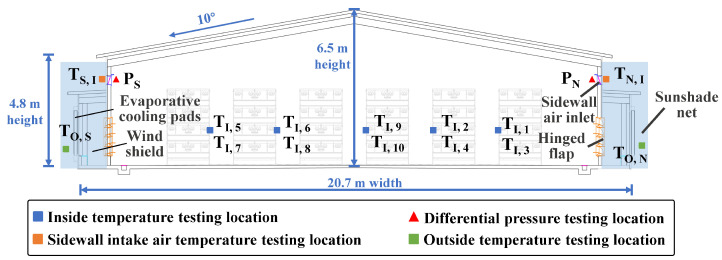
Barn layout and sampling points.

The interior air temperature was monitored from 27 February 2023 to 26 March 2023 to assess the relationship between temperature difference and climate (radiation and velocity). Ten temperature sensors (PT1000, Hotraco Agri, Hegelsom, The Netherlands, Class A accuracy, −50 °C~+150 °C) were used to measure the interior air temperature. As shown in Figure 4 and Figure 5, the temperature dataloggers were installed in the following directions inside the barn to characterize the longitudinal and lateral direction temperature difference profile of the barn: (1) longitudinal direction measurements included two zones with mean values of the temperature sensor T_I, 9_, T_I, 1_, T_I, 5_, T_I, 2_, and T_I, 6_ representing the temperature in the front zone, while the mean values of sensor T_I, 3_, T_I, 7_, T_I, 4_, T_I, 8_, and T_I, 10_ represented the temperature in the rear zone. (2) Along the lateral direction of the barn, the average temperature of sensor T_I, 5_, T_I, 6_, T_I, 7_, and T_I, 8_ represented the temperature on the southern side, whereas the average values of T_I, 1_ T_I, 2_, T_I, 3_, and T_I, 4_ represented the temperature on the northern side. Two temperature dataloggers (Hobo Pro Series Temp/RH logger, Onset Computer Corp., Bourne, MA, USA, ±0.2 °C, −40 °C~+80 °C) were installed on the outer side of the sidewalls’ inlet of the broiler house, with one sensor installed in the middle of the sidewall. Another two temperature dataloggers were installed inside the sunshade net on each side to record the ambient air temperature outside the sidewall during the experimental period. There are two differential pressure sensors (Pressure transducer PrimAtü 10, FSM AG, Kirchzarten, Germany, ±1 Pa, 0~100 Pa) with a range of 0 to 100 Pa for differential pressure, which were set at each sidewall with one for monitoring the differential pressure in the vicinity of the air inlet. Climate conditions such as air temperature, air humidity, wind velocity, and wind direction were recorded by a meteorological observatory (ZK-YD10A Weather station, Zhongke Zhengqi Co., Ltd., Beijing, China). The environmental parameters were recorded at 15 min intervals, while the differential pressure of the air inlet was collected at 1 s sampling intervals. The hourly solar radiation data were provided by the China Meteorological Data Service Centre.

### 2.3. Data and Statistical Analysis

The following statistical analyses were performed by using Excel (Microsoft Office Excel 2021, Microsoft, Redmond, WA, USA) and Python 3 (version 3.9). The measured sensor data at different time levels, including internal temperature data, target temperature data, and climate data, were first converted into hourly average temperatures and then subjected to subsequent analysis to maintain consistency in temporal resolution. The daily means (±SD) of the interior temperatures were analyzed and tabulated for four monitoring periods, categorized according to different ventilation rates: less than 1% (1 fan opened), 1% to 3% (a fan opened), 4% to 7% (2 fans opened), and more than 8% of the total (3 fans opened) (17 February to 26 February, 27 February to 8 March, 9 March to 17 March, and 18 March to 26 March). The air temperature distribution inside the barn was represented by comparing the average temperature of different cross-sections in the longitudinal and lateral directions. The nonuniformity coefficient, defined as the ratio of the standard deviation to the average temperature, is used to evaluate the uniformity of the air temperature distribution in poultry buildings [22]. The Statsmodels library in Python was used to analyze the significant differences in the environmental parameters using ANOVA and Tukey’s Kramer test, with statistical significance set at *p* < 0.05. Due to the deactivation of the heating system, the last testing period was selected for analyzing the relationship between temperature difference and climate conditions. The relationships among the internal environment, climate environment, and differential pressure of slot openings were analyzed by Spearman’s correlation analysis with a coefficient of rank correlation R > 0.40 and *p* < 0.05 indicating a significant correlation [30]. The sign of the coefficient indicates whether it is a positive or negative monotonic relationship. To verify the reasons for the lateral environmental differences in the poultry house, a simple thermal balance model referenced by Wang et al. [31] was developed to illustrate the heat exchange process in the poultry house.

## 3. Results

### 3.1. The Interior Temperature Variation and Distribution during Whole Broiler Production

**Figure 6:**
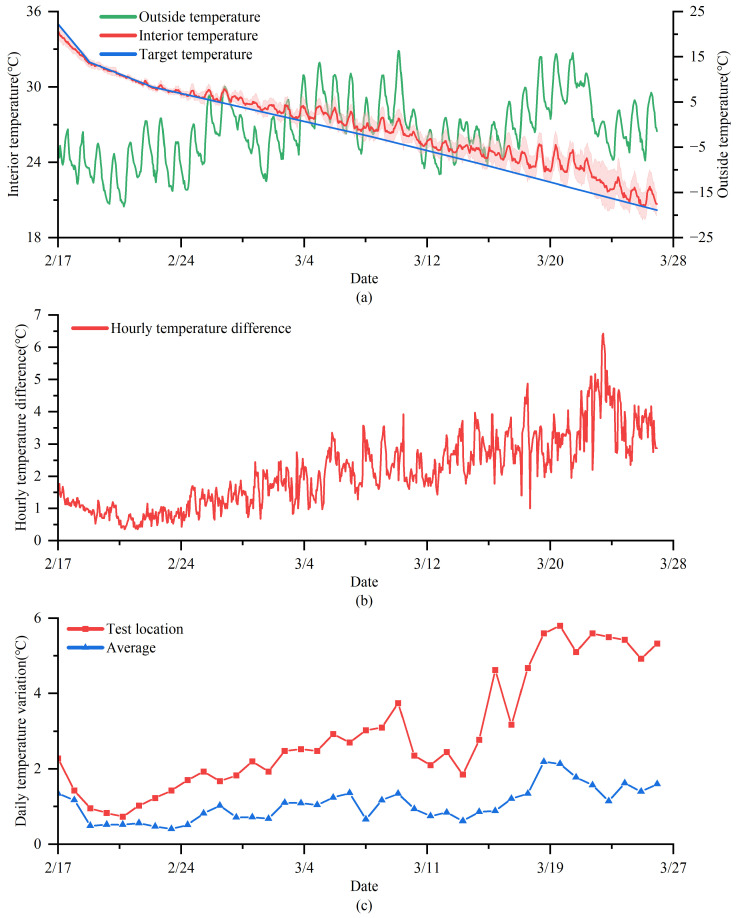
Temperature parameter changes in the equipment during the fattening period: (**a**) hourly interior temperatures, target temperature, and outside temperature; (**b**) hourly temperature difference; and (**c**) daily temperature variation in the test location and average.

The average temperature, temperature difference, and daily temperature fluctuation inside the barn are shown in Figure 6. All measurements of temperature, including interior and outside temperature, were tested simultaneously. As the requirements for broiler temperature changed, the average temperature of various measuring locations within the barn decreased from 33.69 ± 0.59 °C to 21.17 ± 0.50 °C for 38 days of age. During the testing period, the temperature fluctuation outside the poultry house ranged from −12.94 ± 3.79 °C to 9.68 ± 3.84 °C, with a maximum daily temperature difference of 12.71 ± 2.83 °C. The result shows that, as the broiler age increased, the temperature difference between the average interior temperature and the target temperature gradually significantly increased (*p* < 0.001) for the four fattening stages, with values of −0.05 ± 0.26 °C, 0.65 ± 0.38 °C, 0.89 ± 0.39 °C, and 1.29 ± 0.70 °C. Figure 6b illustrates the hourly temperature difference within the barn. As the age of the livestock increases, the temperature difference inside the barn gradually rises. The maximum temperature differences for each testing stage are 1.85 °C, 3.58 °C, 3.98 °C, and 6.43 °C, respectively. Similarly, the daily average temperature at the test point is shown in Figure 3c. The maximum temperature fluctuations of the test locations increased from 2.27 °C to 3.02 °C, then to 4.63 °C, and finally to 5.80 °C as the broiler age increased. The daily fluctuations in the average temperature of the barn were 0.71 ± 0.32 °C, 0.96 ± 0.25 °C, 0.92 ± 0.24 °C, and 1.60 ± 0.36 °C across the four periods, with the average temperature fluctuations being the largest in the last period. Based on the temperature sensor and the heating temperature, the estimated daily heating duration for temperature control in the broiler house during the first three testing periods was 19.6, 12.5, and 14.7 h, respectively.

Table 3 provides a summary of descriptive statistics (mean ± SD) and the results of mean separation analysis for the interior air temperature distribution in the longitudinal and lateral directions during the four monitoring periods, which were predominantly associated with the ventilation rate. During the four testing periods, there was spatial variation in the temperature distribution, indicating that the thermal environment in the barn was not uniform.

In the longitudinal direction, there were significant differences in temperature (*p* < 0.05) between the front and rear areas of the broiler house during the four testing periods. Higher temperatures were consistently recorded at the rear of the barn. And the average temperature of the last testing stage at the rear (23.22 ± 1.84 °C) is warmer than the front (23.03 ± 1.61 °C) by 0.23 °C. The greatest temperature difference between the front and rear locations was measured during the last testing period, with the rear temperature 23.52 ± 0.84 °C warmer than the front temperature 21.66 ± 1.44 °C by 1.87 °C. In the lateral direction, no significant difference (*p* > 0.05) was found in average temperatures measured between the south side (30.66 ± 1.46 °C) and the north side (30.73 ± 1.45 °C) in the first testing stage. However, there are significant differences (*p* < 0.01) in lateral temperatures during the remaining three stages according to the results of Tukey’s honestly significant difference (HSD) test in Table 3. In the fourth testing stage, the maximum temperature difference between the northern and southern measurements is observed, with the temperature on the southern side being 26.14 ± 0.76 °C, which is 2.44 °C higher than the north one 23.70 ± 0.49 °C recorded on the north side. In the fourth farming stage, the temperatures on the south side and the north side both showed significant differences compared to the average temperature inside the barn (*p* < 0.01), which demonstrates a significant temperature nonuniformity in the lateral direction, and the higher daily temperature fluctuations were found on the south side of the poultry house, with average temperature fluctuations of 3.18 ± 0.54 °C compared to 1.91 ± 0.64 °C on the north side during the last period. There were no significant differences in daily temperature fluctuations between the southern and northern locations during the other three testing stages.

**Figure 7:**
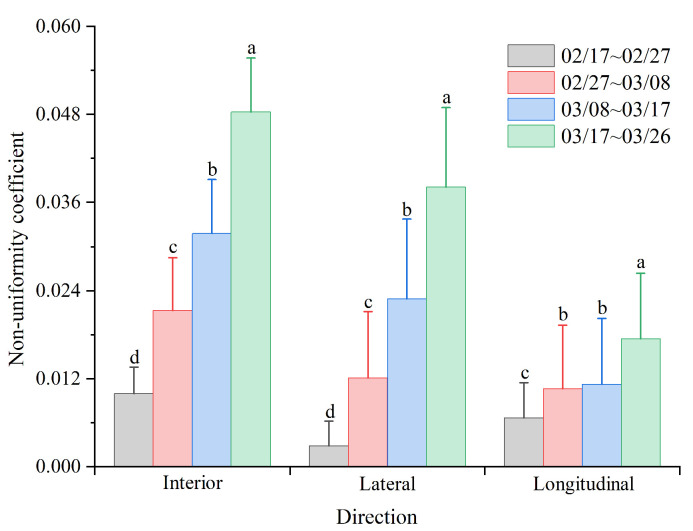
Nonuniformity coefficient of temperature in the overall temperature, longitudinal temperature, and lateral temperature during the four testing stages. The different lowercase letters (a, b, c, and d) within the same direction indicate significant differences between different testing stages, *p* < 0.05, and there were no significant differences.

Figure 7 shows nonuniform distributions of temperature in both the longitudinal and lateral directions inside the poultry house during four testing stages. The picture demonstrates that the temperature uniformity significantly decreased as the broiler age increased (*p* < 0.001). Especially, Tukey’s test results indicate the coefficient of nonuniformity in the lateral direction exhibited a significant increase throughout the four rearing periods (*p* < 0.001) with values of 0.0028, 0.0120, 0.0229, and 0.0381, respectively. However, in the longitudinal direction, there is no significant difference in the coefficient of nonuniformity between the second and third stages (*p* > 0.05). And the coefficient of nonuniformity of the four test stages is 0.0066, 0.0106, 0.0112, and 0.0174, respectively, which was significantly smaller than that in the lateral direction during the third and fourth stages.

### 3.2. Relationship between Temperature Difference and Solar Radiation

Table 4 presents the average descriptive statistical results, encompassing temperature and pressure, for two stages: one with solar radiation and one without. Specifically, the time during which solar radiation is less than 10 MJ/m^2^ is considered the period without solar radiation, while all other periods are categorized as the period with solar radiation. The outside solar radiation (O-SR) during the period with solar radiation was significantly higher than that during the stages without solar radiation (*p* < 0.001), with 459.9 ± 224.2 MJ/m^2^ compared to 1.8 ± 5.0 MJ/m^2^. The average interior temperature in the lateral direction (T-IL) in the southern location (22.30 ± 1.67 °C) was significantly lower than that in the northern location (23.19 ± 1.41 °C) in the absence of solar radiation (*p* < 0.001). However, during periods with solar radiation, the average T-IL in the southern location was slightly higher by 0.23 °C compared to that in the northern location (*p* < 0.05). And during the periods with solar radiation and without solar radiation, the maximum temperature differences on the north and south sides are 2.44 °C and −2.31 °C, respectively. Meanwhile, the temperature of the sidewall inlet (T-SI) and the temperature inside the sunshade net (T-SN) on the south side were warmer than those on the north side (*p* < 0.001), by 1.68 °C and 3.47 °C, respectively, under solar radiation. However, no significant differences (*p* > 0.05) were found in T-SI and T-SN between southern and northern locations without solar radiation.

Table 5 presents the differences in environmental parameters between the north and south sides of the barn house during midday and midnight, corresponding to the period of maximum solar radiation and the absence of solar radiation, respectively. Furthermore, it also corresponds to the times of maximum and minimum temperature differences between the north and south sides throughout the day. At midday, the temperature on the south side of the poultry house was 1.1 °C higher than that on the north side (*p* < 0.001), whereas at midnight, the north side was 1.47 °C higher than that on the south side (*p* < 0.001), indicating a thermal conversion phenomenon on the warmer side of the broiler house. Significant differences in T-IL, T-SI, and T-SN between the south and north sides were noticed (*p* < 0.001), with the difference in average T-IL, T-SI, and T-SN at midday being higher than that at midnight, indicating that the warmer side of T-IL and T-SI changed from the north side to the south side when O-SR increased.

**Figure 8:**
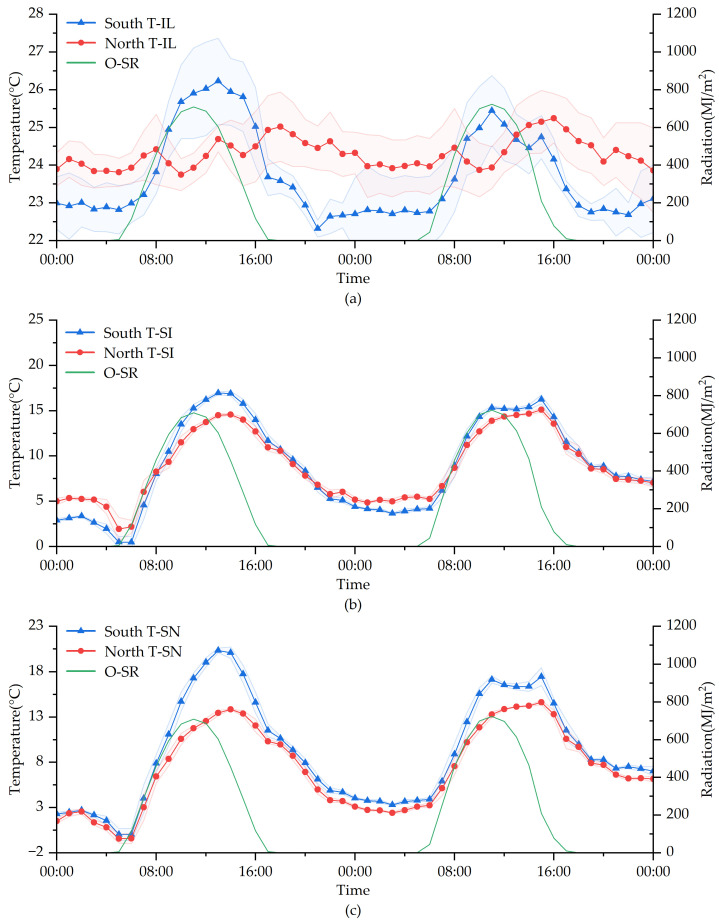
The dynamic variation in the hourly mean temperatures of (**a**) the interior temperature, (**b**) the sidewall inlet temperature, and (**c**) the temperature inside the sunshade net on the south and north sides of the barn in response to solar radiation (O-SR).

Figure 8 presents the typical dynamic relationship between temperature parameters and O-SR in the poultry house during 20 March and 21 March. The results indicated that the difference in T-IL between the southern and northern areas showed a noticeable variation with changes in O-SR. With the absence of solar radiation, the interior temperature on the north side of the broiler house, which was 24.46 ± 0.22 °C, was up to 2.14 °C warmer than the 22.31 ± 0.58 °C observed on the south side. Conversely, with increasing solar radiation, the temperature on the south side of the poultry house could rise by up to 1.98 °C higher than that on the north side, which is 25.91 ± 1.20 °C and 23.93 ± 0.39 °C. Simultaneously, the differences in T-SI and T-SN between the south and north sides exhibited similar trends. With increasing solar radiation, the differences in T-SI changed from −2.52 °C to 2.49 °C, indicating that the T-SI of the south and north locations, respectively, increased from 2.64 ± 0.21 °C and 5.17 ± 0.18 °C to 16.23 ± 0.21 °C and 13.73 ± 0.22 °C. Although there was no significant difference in T-SN between the south and north sides without solar radiation (*p* > 0.05), the increase in solar radiation resulted in a maximum temperature difference of 6.88 °C, with the southern T-SN averaging 20.33 ± 0.26 °C, significantly higher than the northern T-SN average of 13.45 ± 0.29 °C (*p* < 0.01). As the solar radiation increased, the values of T-SI, T-SN, and T-IL on the south side gradually increased, with the temperature on the south side being higher than that on the north side. When the radiation decreased, the southern temperature decreased, ultimately becoming lower than the north. According to Spearman correlation analysis results, the O-SR was strongly positively correlated with the difference in T-IL (R = 0.610 and *p* < 0.001), the difference in T-SI (R = 0.687 and *p* < 0.001), and the difference in T-SN (R = 0.709 and *p* < 0.001). The difference in T-IL was positively correlated with T-SI (R = 0.481 and *p* < 0.001) and T-SN (R = 0.520 and *p* < 0.001).

Based on a simple thermal balance model, a typical case was calculated. On March 20 at 1 PM, the relevant parameters are given in Table 6. The calculated heat loss through the south and north walls due to conduction is 472 W and 901 W, respectively. Assuming that the air inflow rate from both sidewall inlets is the same due to the lack of relevant airflow rate data from both sidewall air inlets. The inlet temperature on the south side is 16.94 °C, which is higher than that on the north side, measured at 14.50 °C. Consequently, the heat loss caused by the air entering through the south and north walls is 408,622 W and 478,620 W, respectively.

### 3.3. Relationship between Difference Temperature and Wind Velocity

Table 4 shows that the average interior temperature in the lateral direction (T-IL) in the southern location (22.30 ± 1.67 °C) was significantly lower than that in the northern location (23.19 ± 1.41 °C) in the absence of solar radiation (*p* < 0.001). Especially, the T-IL on the north side was significantly higher than that on the south side by 1.47 °C (*p* < 0.001) at midnight (Table 5). In Table 2, no significant differences (*p* > 0.05) were found in T-SI and T-SN between southern and northern locations without solar radiation. However, the P-SI on the southern side is significantly higher than that on the northern side (*p* < 0.001).

**Figure 9:**
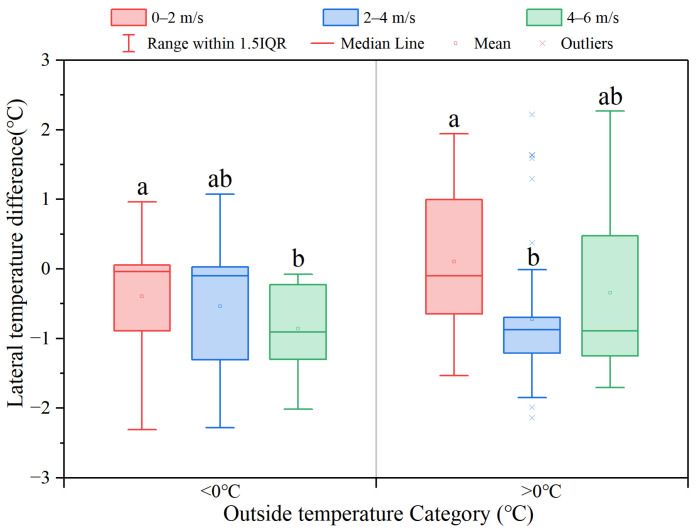
The lateral temperature difference under three wind velocity ranges (0–2 m/s, 2–4 m/s, and 4–6 m/s) and two different outside temperature categories (less than 0 °C and over 0 °C). Different letters “a, b” within the image indicate that there are significant differences in the lateral temperature difference corresponding to different ranges of wind speed (*p* < 0.05) in the same temperature category using the Tukey test for difference of the means.

The daily mean (±SD) wind velocity, which was converted from different directions into the vector perpendicular to the sidewall, was 3.00 ± 2.73 m/s with a range from 0 to 8.87 ± 0.61 m/s. The average wind velocity during the period without solar radiation is 2.40 ± 2.11 m/s, and Spearman correlation analysis results showed that the wind velocity was strongly correlated with the difference in P-SI, with R = 0.814 and *p* < 0.001. Also, the Spearman correlation analysis results indicate strong coupling between O-SR and wind speed outside the poultry house (R = 0.529 and *p* < 0.001). Therefore, the analysis of wind speed’s impact on the lateral temperature difference was performed without solar radiation. Figure 9 shows the relationship between the lateral temperature difference and wind speed ranges. When the outdoor temperature was below 0 °C, there was no significant difference in the lateral temperature difference between range 1 (0–2 m/s) and range 2 (2–4 m/s), but it was significantly higher than that in range 3 (4–6 m/s) (*p* < 0.05). Similarly, when the outdoor temperature was above 0 °C, the temperature difference in range 1 was significantly greater than that in range 2 (*p* < 0.001).

### 3.4. Productivity and Viability of Broilers

Table 7 presents the broiler’s mortality rate and performance at the four growing stages. Comparisons of the recorded data showed that mortality rates were not significantly different between the first and second stages. But the average daily mortality rate of the fourth stage is significantly higher than that of the first three fattening stages (*p* < 0.05), with 1.46 ± 0.27‰ compared to 0.81 ± 0.18‰, 0.54 ± 0.13‰, and 0.49 ± 0.16‰. According to the average daily weight gain data in Table 7, there are no significant differences in daily weight gain data among the last three fattening stages. However, the difference in weight gain compared to the standard performance for the last breeding stage, as provided by Aviagen (2022) (Huntsville, AL. USA), is greater than in the previous three stages, with −15 g/bird comparing to −4.9 g/bird, 2.9 g/bird, and −2.0 g/bird. (The negative sign indicates that the daily weight gain is lower than the standard value.) The average daily weight gain and daily intake show an increasing trend with the increase in age, which is similar to the growth curve of the AA+ (Arbor Acres Plus) broiler breed standard performance. As the age of the broiler chickens increases, the average daily FCR (feed conversion ratios: weight gain ÷ feed intake) gradually increases, and the average FCRs for the four stages are 1.18, 1.24, 1.60, and 1.99. The average FCR of the broilers throughout the entire fattening stage is 1.55 compared with the standard value of 1.496. During the second testing stage, the FCR of the broilers is 0.1 lower than the standard value. At other testing periods, the FCR of the broilers is higher than the standard value, with the highest FCR in the last fattening stage at 1.99, which is 0.2 higher than the standard value of 1.79.

## 4. Discussion

In this study, we monitored the air temperature distribution throughout the entire broiler house in winter. As the age of the broilers increased, the temperature decreased from 33.69 ± 0.59 °C to 21.17 ± 0.50 °C in accordance with the temperature requirements of the broilers. Meanwhile, the daily temperature fluctuations within the house and the temperature control errors have been gradually increasing; a similar temperature pattern was reported by Wheeler et al. [17]. One of the most important reasons is that the use of heaters within the poultry house gradually decreases with the age of the poultry, which causes the indoor environment to become more susceptible to the external environment [32]. The temperature fluctuated significantly during the first two days of broiler chicks’ life, where the main reason was the large variations in the target temperature of the poultry house, as shown in Figure 6a, which led to deviations in the environmental control of the house. During the broiler farming process, the average daily heating period in the first testing period was longer due to a higher target temperature. Although the target temperature in the third period was lower than that in the second period, the heating duration in the third stage was actually higher than second period, which was attributed to the lower outdoor temperature during third period.

### 4.1. The Uniformity of Interior Temperature

There was no significant temperature difference in the lateral direction during the first testing stage (*p* > 0.05). This finding is consistent with the results reported by Wheeler et al. because the outside surroundings and chicks with less heat and moisture production had little effect on the interior environment during the early rearing days [17]. However, during the first two days of broiler chicks’ life, the indoor temperature exhibited significant temperature differences, which may primarily be attributed to variations in target temperature, leading to deviations in temperature control. In the last testing stage, with the increase in age and ventilation rate, there is a significant temperature difference, as shown in Figure 6b, and the lateral temperature nonuniformity increases with the age of broilers, which could be attributed to external environmental factors or uneven ventilation of the barn [31].

For the longitudinal direction, the lengthwise temperature stratifications remained persistent throughout the following grow-out period, that is, the warmer location near the fans (Table 3). Zheng et al. noted a similar pattern of temperature distribution along the building length direction in a poultry house [33]. This is due to a gradual accumulation of thermal energy along the direction of air exhaust within the poultry house, which results in a higher temperature at the rear of the house [15,25,34]. As the broiler age increased, the nonuniformity in the longitudinal direction increased with bird age because of the larger heat production of broilers [35], but there was no significant difference in the lengthwise temperature between the second and third testing periods. The ventilation rate was increased in the housing systems, resulting in weakened longitudinal temperature stratification (Figure 7).

In this paper, we concentrated on monitoring the temperature distribution in both vertical and horizontal directions, while neglecting the temperature distribution in the vertical direction. In previous studies, we found that the temperature in the upper cages is higher than that in the lower cages in stacked-deck hen houses when the tested cages are located in the middle row of the house [33]. The main reason is that heat always tends to accumulate in higher spaces [17]. In addition, Ma et al. monitored the broiler houses with the same ventilation mode and found that in a row of chicken cages near the side walls, the temperature of the bottom layer of the cages was higher than that of the upper layer. This was due to the cold air coming in from outside, which caused a decrease in temperature in the upper cages of the poultry house [22]. Moreover, as the age of broilers increases, an increase in ventilation will reduce the temperature stratification within the house [34].

### 4.2. The Effect of Solar Radiation on the Interior Temperature Difference

In the lateral direction, due to the relative symmetry of the poultry house structure and ventilation system in the lateral direction, most studies have found a relatively uniform transverse temperature distribution because of the uniform ambient conditions and ventilation parameters on both sides of the broiler house [14,36,37]. But the monitoring results indicate that the average T-IL in the southern location is warmer than the northern under solar radiation during the final testing stage, and the T-SI and T-SN on the south side are also higher than those on the north side (*p* < 0.001). One of the reasons for the temperature difference in the barn is the nonuniform solar radiation on the north and south sides. Due to the north–south direction orientation of the building, the south sidewall, air, and roof absorb more heat during the period with solar radiation compared to the northern sidewall [31]. However, according to the heat transfer calculation results, the difference in thermal conductivity through the north and south sidewall of the poultry house is very small, at 429 W. In contrast, due to the differences in temperature and humidity of the intake air on the north and south sidewalls, the enthalpy values of the south sidewall airflow are higher than the north sidewall, so the ventilation heat loss from the south side is 70,427 W smaller than that from the north side. Therefore, solar radiation primarily affects the inlet air temperature on the sidewalls of the poultry house, which in turn influences the ventilation heat exchange of the poultry house, thus affecting the lateral temperature differences inside the house [38,39,40]. A higher difference in T-SI between the south and north sidewalls would potentially result in greater heat convection transfer, which is the main reason that the T-IL on the south side is higher than that on the north at midday, and as the solar radiation decreases, the heat absorbed by the southern sidewall also reduced, causing the T-SI and T-IL to decrease. It should be noted that the main purpose of the calculation is to verify the influence of wall heat conduction and ventilation heat exchange influenced by solar radiation; therefore, the effect of external wind speed on the air inflow volume from both side walls is neglected. However, based on the temperature distribution in the poultry house, we can still infer that the effect of solar radiation on the temperature difference (inlet temperature) is greater than the influence of wind speed (inlet volume) when solar radiation is intense.

### 4.3. The Effect of Outside Wind Velocity on the Interior Temperature Difference

The northern side (23.19 ± 1.41 °C) exhibits a significantly higher T-IL than the southern side (22.30 ± 1.67 °C) without solar radiation, and the T-IL on the north side was significantly higher than that on the south side by 1.47 °C (*p* < 0.001) at midnight (Table 5). Moreover, there is no significant difference in T-SN and T-SI between the north and south sides with the reduction in solar radiation. However, the P-SI on the southern side is significantly higher than that on the northern side, with an average of 1.8 Pa during the period without solar radiation and 2.1 Pa at midnight. P-SI is sensitive to the velocity of the incoming air, and the airflow is driven by the pressure difference across the slot openings [41]. The inlet airflow rate is presented as a function of P-SI with the same opening of the sidewall inlet [42]. The airflow of the sidewall inlet is the main heat convection method to transfer energy inside a ventilated building in winter [43,44]. Consequently, the southern side experiences a higher ventilation rate of cooler air, which is the main reason for the lower T-IL on the southern side compared to the northern side.

In Figure 9, the temperature difference in wind speed range 1 was significantly greater than that in range 2 when the outdoor temperature was above 0 °C, and the temperature in wind speed 3 was higher than range 1 and range 2 when the outdoor temperature was below 0 °C, which indicated that there was a tendency for an improvement in the lateral temperature difference as the wind load on the southern sidewall increased. Furthermore, as the wind speed increased, the airflow rate of cold air on the south side increased, resulting in lower temperatures on the south side. The reason for the higher P-SI on the southern side is that the south side is the windward side. The source of pressure difference in the sidewall inlet includes negative pressure force by gable wall fans and wind force [28,45,46,47]. According to existing research, the pressure driven by the wind was positively correlated with the air velocity, and the results of the Spearman correlation analysis between wind speed and P-SI followed this pattern (R = 0.529) [48]. This conclusion could be easily demonstrated through the calculation of the thermal balance equation of the chicken house [26]. On the other hand, the higher wind speed on the southern side may result in a larger convective heat transfer coefficient, leading to increased heat dissipation through the southern wall [49]. Therefore, during the period with solar radiation, the walls, roof, and air on the southern side are heated by solar radiation, which were the main factors causing the higher interior temperature on the southern side, while the higher inlet air volume and heat transfer coefficient on the southern side caused by the outside wind speed resulted in a higher temperature on the northern side without solar radiation.

### 4.4. The Productivity and Viability of Broilers

In the first testing stage, the mortality rate and FCR of the broiler chickens were lower than in the other three periods. During this period, the temperature fluctuations and nonuniformity inside the house were smaller, because the low ventilation rate and the heating equipment used made the indoor environment comfortable for the broilers. As the age of the broiler chickens increased, their mortality rate and feed conversion ratio gradually increased, especially during the third and fourth testing periods. In the third testing period, the mortality rate was significantly higher than in the first two testing periods, and the daily weight gain was lower than the standard value while the feed intake was above the standard level. As shown in Figure 3, although the average temperature fluctuations during this stage are relatively small, the temperature inside the house is uneven. Additionally, there are significant temperature fluctuations at certain test locations, which may result in poor production performance of the poultry indoors [4]. The highest mortality rate and FCR occurred in the final period, with an average daily mortality of 70 to 80 broilers. One potential reason is that the high-density rearing method can affect the health and performance of broilers, as the increased body weight reduces their activity space in the cages and worsens their welfare conditions [50,51]. Another important reason is that broiler houses are more susceptible to the influence of external environmental fluctuations because of the heater being turned off, leading to substantial variations in the average temperature, especially in the lateral direction of the houses. The temperature fluctuations and temperature uniformity during the fourth period are significantly worse than those during the previous three periods. At the same time, the mortality rate and feed conversion rate of broilers also show similar trends. It is evident that the indoor environment has a substantial impact on the production performance of broilers. Some studies have recommended that the fluctuation in house temperature may lead to a poor daily body weight feed conversion ratio and to health problems for poultry [14,52]. On the other hand, the nonuniformity of temperature results in lower temperatures in certain areas while higher temperatures in others inside the broiler house. High temperatures would result in lower feed intake, lower body weight, and higher feed conversion ratio of poultry, while low brooding temperatures have been shown to increase ascites mortality [12,53]. The environment, as an important influencing factor, determines the profitability of broiler farming in farm production. If the environment of the poultry house can be well controlled, it can improve the production performance of broilers to near standard values, bringing significant economic benefits to the farm.

To reduce the impact of the outdoor climate on the indoor environment, it is important to consider both wind direction and the angle of solar radiation when designing the orientation of the broiler house. This will help alleviate their effects on the indoor environment. The poultry house should be oriented in an east–west direction [54]. To reduce the impact of uneven solar radiation from outside on the indoor environment, the uniformity of indoor temperature can be improved by optimizing the building’s insulation performance [55]. Measures can be implemented to shield the air inlets on the sidewalls of the chicken house in order to reduce the directly impact of external wind speed. A new ventilation system proposed by Wang et al. involves installing buffer chambers outside the sidewalls of the chicken house, which can improve the uniformity of the indoor temperature effectively [34]. Typically, once the poultry house has been built, it is not easy to optimize or adjust the ventilation system, insulation performance, and orientation of the poultry house. Based on the heat transfer ways of the poultry house, we can adjust the airflow rate from both sides of the chicken house to mitigate the lateral temperature difference within the house by controlling the angle of the sidewall air inlets [56]. For example, when excessive heat loss due to ventilation at the north sidewall inlet results in a lower temperature on the north side, it is possible to reduce the temperature difference in the poultry house by adjusting the angle of the north sidewall inlet to minimize the inflow of cold air. At the same time, when the angle of control changes, parameters such as the velocity of airflow, the volume of intake air, and the pressure difference on both sides of the inlet will also vary accordingly. The relevant patterns need to be further studied in order to improve the uniformity of the poultry house environment through the control and management of the ventilation system.

## 5. Conclusions

The following conclusions were drawn from the assessment of interior temperature differences in a commercial stacked-deck cage broiler house. (1) The hourly average temperature inside the barn decreased from 33.69 ± 0.59 °C to 21.17 ± 0.50 °C at 38 days of age. The maximum temperature difference increased from 1.85 °C to 6.43 °C, while the daily fluctuation increased from 2.27 °C to 5.80 °C, which indicated that the uniformity and stability of temperature decreased as the broiler’s age increased. (2) The average T-IL at the southern location was 1.10 °C higher than that at the northern location, mainly because solar radiation heats the air near the south sidewall inlet, resulting in a higher enthalpy of the air entering the south location of the barn. (3) The average T-IL of the southern location was 1.47 °C lower than that of the northern location at midnight, which was due to the P-SI in the north sidewall being 2.1 Pa lower than that in the south sidewall because of the outside wind speed, resulting in increased cold air intake volume into the southern side of the broiler house. This study has shown that the poor temperature environment caused by solar radiation and outside wind can affect the production performance of broilers. It is suggested that when managing and controlling the poultry house environment, attention should be paid to the impact of the external climate.

## Figures and Tables

**Table 1:** Types and dimensional parameters of the fattening systems.

Fattening System	Description	Dimensions (m) ^1^
Cage	Conventional cage system	2.4 × 1.8 × 0.65 (length, width, and height, per cage)
Drinker	Nipple-type drinker	/
Feeder	Round feeder pan	0.32 (diameter)
Manure removal	Conveyor belt	83.0 × 1.8 (length and width)

^1^ This column provides the dimensional parameters for each cage, feed tray, and conveyor belt.

**Table 2:** The ventilation and temperature control of broiler house.

Broiler Age	0	1	3	7	14	21	28	35	38
Min vent (m^3^/h)	3280	5110	7850	10,800	17,970	27,730	44,930	53,470	60,020
Max vent (m^3^/h)	10,000	10,000	20,000	40,000	70,000	120,000	190,000	270,000	300,000
Heat temp (°C)	34.2	34	31.7	29.7	27.7	25.7	23.7	21.2	19.9
Cooling temp (°C)	/	/	/	/	33	31	29	26.5	25.2

**Table 3:** The average temperatures inside the poultry house and in different directions.

Testing Period	Interior Temperature	Lateral Direction		Longitudinal Direction	
		North	South	Front	Rear
17 February~26 February	30.69 ± 1.44 ^a, AB^	30.66 ± 1.46 ^a^	30.73 ± 1.45 ^a^	30.58 ± 1.40 ^B^	30.80 ± 1.47 ^A^
27 February to 8 March	28.07 ± 1.00 ^b, C^	28.08 ± 0.92 ^b^	28.22 ± 0.93 ^a^	27.98 ± 0.99 ^B^	28.16 ± 0.99 ^A^
9 March~17 March	25.63 ± 1.20 ^b, AB^	25.96 ± 0.85 ^a^	25.61 ± 1.39 ^b^	25.57 ± 1.20 ^B^	25.69 ± 1.19 ^A^
18 March to 26 March	23.12 ± 1.74 ^c, AB^	23.44 ± 1.46 ^a^	22.93 ± 1.84 ^b^	23.03 ± 1.61 ^B^	23.22 ± 1.84 ^A^

^a, b, c^ Different superscript lowercase letters within the same testing period indicate that means differ significantly between temperature in the lateral direction and interior average temperature (*p* < 0.05) using the Tukey test for difference of the means. ^A, B, C^ Different superscript uppercase letters indicate the significant difference between temperature in the longitudinal direction and interior average temperature.

**Table 4:** The mean value of the interior temperature in the lateral direction (T-IL), temperature of the sidewall inlet (T-SI), temperature inside the sunshade net (T-SN), static pressure difference between both sides of the sidewall inlet (P-SI) during the period with and without solar radiation.

Time	Location	T-IL ^2^ (°C)	T-SI (°C)	T-SN (°C)	P-SI (Pa)
Period without solar radiation	North	23.19 ± 1.41 ^a^	3.67 ± 5.43 ^a^	2.50 ± 5.10 ^a^	19.2 ± 5.9 ^b^
	South	22.30 ± 1.67 ^b^	3.23 ± 5.67 ^a^	3.07 ± 5.53 ^a^	22.0 ± 6.4 ^a^
Period with solar radiation	North	23.35 ± 1.38 ^b^	7.01 ± 6.05 ^b^	6.31 ± 6.06 ^b^	18.1 ± 7.1 ^b^
	South	23.58 ± 1.97 ^a^	8.69 ± 5.93 ^a^	9.78 ± 6.54 ^a^	24.7 ± 6.2 ^a^

^a, b^ Different superscript lowercase letters within the same time and the same parameter indicate that means differ significantly (*p* < 0.05) using the Tukey test for the difference of the means.

**Table 5:** Summary of descriptive statistics (mean ± SD) and the difference in the mean separation analysis for temperature and pressure difference between the south and north sides at midnight and midday ^1^.

Time ^2^	T-IL (°C)	T-SI (°C)	T-SN (°C)	P-SI (Pa)
Midnight	−1.47 ± 0.84 ^b^	−0.74 ± 0.77 ^b^	0.46 ± 0.57 ^b^	2.1 ± 2.4 ^b^
Midday	1.10 ± 0.84 ^a^	3.12 ± 2.90 ^a^	5.85 ± 3.50 ^a^	8.0 ± 5.8 ^a^

^1^ The lateral temperature and pressure difference is calculated by subtracting the average temperature or pressure on the north side from the south side. A larger temperature of the pressure difference indicates a higher value on the south side. When the temperature of the pressure difference is greater than 0, the value on the south side is higher than that on the north side. Conversely, when it is less than 0, the temperature on the north side is higher than that on the south side. ^2^ The midday and midnight time represented the presence and absence of solar radiation, respectively, with midday referring to the time range of 11:00–14:00 and midnight referring to the time range of 23:00–2:00. ^a, b^ Different superscript lowercase letters within the same parameter indicate that means differ significantly (*p* < 0.05) using the Tukey test for the difference of the means.

**Table 6:** Parameters for calculating heat transfer in poultry houses.

Location	T-IL (°C)	T-SI (°C)	H-SI (%)	T-SN (°C)
South	26.23	16.94	35.75	20.33
North	24.69	14.50	38.37	13.45

**Table 7:** The average performance of broilers in four different stages.

Indicator	Date			
	17 February~26 February	27 February to 8 March	9 March~17 March	18 March to 26 March
Mortality rate ^1^ (‰ day^−1^)	0.49 ± 0.16 ^c^	0.54 ± 0.13 ^c^	0.81 ± 0.18 ^b^	1.46 ± 0.27 ^a^
Weight gain (g/bird)	26.1 ± 12.3 ^b^	63.4 ± 17.9 ^a^	81.4 ± 14.9 ^a^	83.0 ± 35.4 ^a^
Standard weight gain ^2^ (g/bird)	28.1 ± 8.8	60.5 ± 10.1	86.3 ± 6.0	98.0 ± 1.5
Feed intake (g/bird)	30.8 ± 12.1 ^d^	78.9 ± 16.2 ^c^	130.1 ± 16.4 ^b^	164.8 ± 4.9 ^a^
Standard intake (g/bird)	29.9 ± 11.7	75.8 ± 16.3	129.3 ± 15.7	175.9 ± 12.3
Feed conversion ratios	1.18	1.24	1.60	1.99
Standard feed conversion ratios	1.06	1.25	1.49	1.79

^1^ Mortality rate is the ratio of daily mortality poultry to the total rearing quantity. ^2^ The standard weight gain and standard intake were provided by Aviagen (2022). ^a–d^ The different superscript letters (“a”, “b”, “c”, and “d”) within the same indicator indicate significant differences for a different stage, *p* < 0.05 level, and there were no significant differences.

## Data Availability

Data will be made available on reasonable request from the corresponding author.
